# The effect of bearing and rearing a child on blood pressure: a nationally representative instrumental variable analysis of 444 611 mothers in India

**DOI:** 10.1093/ije/dyab058

**Published:** 2021-07-19

**Authors:** Felix Teufel, Pascal Geldsetzer, Nikkil Sudharsanan, Malavika Subramanyam, H Manisha Yapa, Jan-Walter De Neve, Sebastian Vollmer,, Till Bärnighausen

**Affiliations:** 1 Heidelberg Institute of Global Health (HIGH), Faculty of Medicine and University Hospital, Heidelberg University, Heidelberg, Germany; 2 Division of Primary Care and Population Health, Department of Medicine, Stanford University, Stanford, CA, USA; 3 Indian Institute of Technology, Gandhinagar, Gujarat, India; 4 Kirby Institute, University of New South Wales, Sydney, Australia; 5 Department of Economics, University of Goettingen, Goettingen, Germany; 6 Centre for Modern Indian Studies, University of Goettingen, Goettingen, Germany; 7 Africa Health Research Institute (AHRI), Somkhele and Durban, South Africa; 8 Harvard Center for Population and Development Studies, Harvard University, Cambridge, MA, USA

**Keywords:** Blood pressure, pregnancy, child-rearing, instrumental variable analysis, women’s health, global health

## Abstract

**Background:**

At the individual level, it is well known that pregnancies have a short-term effect on a woman’s cardiovascular system and blood pressure. The long-term effect of having children on maternal blood pressure, however, is unknown. We thus estimated the causal effect of having children on blood pressure among mothers in India, a country with a history of high fertility rates.

**Methods:**

We used nationally representative cross-sectional data from the 2015–16 India National Family and Health Survey (NFHS-4). The study population comprised 444 611 mothers aged 15–49 years. We used the sex of the first-born child as an instrumental variable (IV) for the total number of a woman’s children. We estimated the effect of an additional child on systolic and diastolic blood pressure in IV (two-stage least squares) regressions. In additional analyses, we stratified the IV regressions by time since a mother last gave birth. Furthermore, we repeated our analyses using mothers' husbands and partners as the regression sample.

**Results:**

On average, mothers had 2.7 children [standard deviation (SD): 1.5], a systolic blood pressure of 116.4 mmHg (SD: 14.4) and diastolic blood pressure of 78.5 mmHg (SD: 9.4). One in seven mothers was hypertensive. In conventional ordinary least squares regression, each child was associated with 0.42 mmHg lower systolic [95% confidence interval (CI): –0.46 to –0.39, *P *<* *0.001] and 0.13 mmHg lower diastolic (95% CI: –0.15 to –0.11, *P *<* *0.001) blood pressure. In the IV regressions, each child decreased a mother’s systolic blood pressure by an average of 1.00 mmHg (95% CI: –1.26 to –0.74, *P *<* *0.001) and diastolic blood pressure by an average of 0.35 mmHg (95% CI: –0.52 to –0.17, *P *<* *0.001). These decreases were sustained over more than a decade after childbirth, with effect sizes slightly declining as the time since last birth increased. Having children did not influence blood pressure in men.

**Conclusions:**

Bearing and rearing a child decreases blood pressure among mothers in India.

**Key Messages**

- Bearing and rearing a child decreases blood pressure among mothers in India.
- As fertility continues to decline in India and similar countries, policy makers should design and implement hypertension screening and prevention programmes that specifically target women.
- Novel health programmes beyond antenatal care are needed to ensure that women without children receive blood pressure screening.

## Introduction

Pregnancy induces substantial changes in a woman’s cardiovascular system.^1^ Towards delivery, systolic and diastolic blood pressure increase by >5 mmHg,^2^ and ∼10% of pregnant women in India develop gestational hypertension or pre-eclampsia.^3^ Throughout motherhood, women also experience considerable changes in their lifestyles and health behaviours, such as altered physical activity levels, diets, sleep patterns and stress exposures.^4^ In India, despite a recent decrease, fertility rates have been high during the last decades and remain high in several states.^5^ At the same time, the country is confronted with a rapidly growing burden of hypertension and cardiovascular disease (CVD).^6^^,^^7^ CVD on average manifests more than 5 years earlier among Indians compared with other populations,^8^^,^^9^ and a large share of CVD morbidity and mortality in India occurs among premenopausal women.^10^ Taken together, these findings raise concerns that bearing and rearing a child might persistently raise blood pressure and eventually increase hypertension and CVD risk among mothers in India.^11^

Past cross-sectional^12–18^ and longitudinal studies^19–22^ that examined the association of women’s blood pressure and the number of children have generated inconsistent findings and have not been population-representative. Moreover, they have been prone to biases due to unobserved confounding that cannot be eliminated by standard statistical adjustments.^23^^,^^24^ Various complex behavioural,^25^^,^^26^ biologic^27^^,^^28^ and socio-demographic factors^29^^,^^30^ are likely to confound the association of blood pressure and the number of children. For instance, psychological traits leading to risk-taking behaviours among women may lead to both additional (unintended) pregnancies and unfavourable health behaviours affecting blood pressure, hence confounding the association.^25^^,^^26^ Thus, it remains unknown whether bearing and rearing a child yields a long-term causal effect on maternal blood pressure. A randomized experiment would eliminate confounding biases but is of course not feasible for major life choices such as childbirth.

Instrumental variable (IV) analyses are a powerful approach for causal inference, which can be used when randomized controlled trials are not possible.^23^^,^^31^^,^^32^ They exploit random variation in nature or due to policy or human practices for confounding control. We used the sex of the first-born child as such a source of random variation. Whereas the sex of the first child is determined at random, it can itself influence future fertility choices. In countries with a strong son preference, such as India, women are often motivated to pursue further pregnancies until the birth of a first boy.^33–35^ The sex of the first child is thus a highly plausible IV to identify random variation in the number of children among women in India.^34^^,^^35^ We used this IV approach to estimate the long-term causal effect of having an additional child on blood pressure among mothers in India.

## Methods

### Data source

We used the National Family and Health Survey (NFHS-4) conducted in India between 2015 and 2016 as the data source for our study. The NFHS-4 provides cross-sectional nationally representative data on women aged 15–49 years for all 29 Indian states, 7 union territories and 640 districts. It employs a stratified two-stage sample design utilizing the 2011 census as a sampling frame. Primary sampling units were census enumeration blocks (in urban strata) and villages (in rural strata). From each of the 28 586 selected primary sampling units, 22 households were randomly selected via systematic random sampling. The NFHS-4 had response rates of 96% for households and 95% for mothers.^36^

### Study population

Our study population comprised all mothers aged 15–49 years who (i) did not report a current pregnancy, (ii) did not give birth to twins at first pregnancy, and (iii) completed the blood pressure measurement in the NFHS-4. We excluded currently pregnant women (*n *=* *19 693), as our objective was to measure the long-term effects of bearing and rearing a child rather than the well-known short-term hemodynamic effects of pregnancy.^2^ Mothers who gave birth to twins at first birth (*n *=* *2672) needed to be excluded because they did not experience a latency period between the birth of the first and the planning of the second child. Lastly, we excluded a total of 9643 mothers (2.1% of the remaining sample) who were missing blood pressure measurements. Supplementary Figure S1, available as Supplementary data at *IJE* online, shows a study-participant flow diagram. The response rate among selected women was near universal (95%) and we only excluded a very small share of these women due to missing data (2.1%). The final sample for our main analyis comprised 444 611 mothers aged 15–49 years.

### Dependent and independent variables

Our exposure was the self-reported number of children beyond the first child, no matter whether a child was alive at the time of the interview or not. Our key outcomes were continuous systolic and diastolic blood pressure. Blood pressure was measured three times for each woman using an Omron Blood Pressure Monitor with a time interval of 5 minutes between readings.^36^ We took the average of all three measurements.^37^ If measurements were missing (2.6% of our sample for the main analysis were missing one and 1.3% were missing two measurements), we used the remaining measurements to calculate individual blood pressure.

### Identification strategy

Our main question was whether bearing and rearing a child has a long-term causal effect on blood pressure in mothers. To draw causal inferences about the effect of children on maternal blood pressure, we employed an IV study design. Under the assumptions described below, this quasi-experimental method can account for unobserved confounding^23^ and thus generate internally valid effect size estimates.^38^ The nationally representative nature of our data ensures high external validity of the effect sizes.

An instrumental variable (IV) is a variable that is associated with the exposure of interest, but is independent of observed and unobserved confounders and does not relate to the outcome other than through the exposure.^39^ In our study, the IV or *instrument* was the sex of a woman's first-born child (Supplementary Figure S2, available as Supplementary data at *IJE* online). This instrument has previously been used to analyse the effect of having children on economic or demographic outcomes among study populations in India and other countries.^33^^,^^40^^,^^41^ Those studies also provide evidence in support of the validity of the instrument. The determination of fetal sex can be compared with a coin flip, where the sex of the first-born child is unlikely to be associated with maternal characteristics. Son preference, which is pervasive in India,^34^^,^^35^ encourages women with first-born girls to pursue further pregnancies until the birth of a boy. As shown in this and the aforementioned studies, son preference thus induces an increased total number of children in mothers with first-born girls.^33^^,^^40^ Taken together, the biological fact that a child’s sex is random and the behavioural fact that the sex of the first-born child affects the total number of children generate the opportunity for IV analysis to estimate the causal effect of having an additional child on maternal blood pressure.

### Statistical analyses

In addition to presenting descriptive sample characteristics, we show the geographic distribution of our exposure and outcomes. We calculated the mean number of children and systolic and diastolic blood pressure levels of mothers in our sample across all 640 districts from the 2011 census in India.^42^ We measured spatial autocorrelation of our exposure and outcomes using Moran’s I.

To measure the causal effect of bearing and rearing an additional child beyond the first on maternal blood pressure, our analysis followed the common structure of IV analyses and included four estimations: (i) conventional ordinary least squares (OLS) regression; (ii) reduced-form regression; (iii) first stage of a two-stage least squares (2SLS) regression; and (iv) second stage of the same 2SLS regression.^24^^,^^38^^,^^43^ Whereas conventional OLS estimates might allow a first assessment of the observed relationship, they are prone to confounding bias, thus motivating the implementation of an IV approach. In the so-called reduced-form regression,^39^ which is analogous to an intention-to-treat analysis in a randomized controlled trial, we directly regressed maternal blood pressure on the instrument. The corresponding estimate is the effect of our instrument (i.e. the sex of the first child) on our outcomes (i.e. maternal systolic and diastolic blood pressure). We show these results in Supplementary Figure S3, available as Supplementary data at *IJE* online. Next, in the first stage of the 2SLS regression, we regressed the number of children on the instrument. The first-stage estimate reveals how the sex of the first-born child influences, on average, the overall number of children. Lastly, in the second stage of the 2SLS regression, we included the predicted number of children from the first stage as an independent variable in a linear regression with maternal systolic and distastolic blood pressure as the outcomes. The resulting effect size represents the causal effect of each child on maternal blood pressure, estimated in the population of women who pursue a further child if their first-born child was a girl, i.e. the so-called complier average causal effect.^38^^,^^39^ The model equations for the four different regression analyses can be found in the Supplementary Methods, available as Supplementary data at *IJE* online. 2SLS regressions were calculated using the Stata command ‘ivregress’. When we used the limited-information maximum-likelihood estimator instead of the 2SLS estimator for the IV estimation, our results remained essentially the same.

### Covariates

The inclusion of covariates is not necessary for unbiased causal inference in IV analyses.^24^ However, covariates can increase the precision of estimates and strengthen the case for the IV by adjusting for potential violations of the IV assumptions.^38^ As is customary in IV estimation, we thus included a range of covariates in our main analysis: age, household wealth (quintiles of the first component estimated in a principal component analysis of household assets) and years of education. We also estimated models with additional covariates and a model without covariates as sensitivity analyses.

### Effect heterogeneity

To measure the change in the effect of bearing and rearing a child on maternal blood pressure over time following the birth of a child, in an additional analysis, we stratified our estimation by the time that had passed since the last time a woman had given birth. We divided our sample into three groups of equal size according to the number of months passed since the last birth. We rounded group cut-offs to the closest year.

### Analyses in men

In a subsample of 15% of households in the NFHS-4, blood pressure was measured among men aged 15–54 years.^36^ We linked these men to the birth histories of their wives or partners. This allowed us to conduct an IV analysis for men in the data set. The rationale behind this analysis is to further elucidate the potential biological and behavioural factors driving effects in mothers. The biological effects of pregnancy and some of the behavioural effects of child bearing and rearing are specific to mothers, while other behavioral effects of child bearing and rearing are observed in both mothers and fathers.^22^ In these analyses, we applied the same exclusion criteria and model specifications as in the analyses in women.

### Support for the instrumental variable assumptions

The key advantage of the IV approach is that, as long as the assumptions for a valid instrument are met, it generates unbiased effect estimates, just like a randomized controlled trial.^23^^,^^31^^,^^32^ The main IV assumption is the *exclusion restriction* that there must be no relationship between the IV and the outcome other than through the exposure. Supplementary Text S1, available as Supplementary data at *IJE* online, discusses the IV assumptions in detail and explains why they are likely met in our analysis. We empirically assessed the IV assumptions using the following tests. First, we assessed whether relevant observed variables varied across the two values of the instrument. This test is akin to the balance test in a randomized controlled trial.^23^^,^^32^^,^^38^ If observed variables did vary across instrument values, this would raise concerns that our instrument is associated with unobserved confounders. Second, we explored whether sex-selective abortions (as implied by birth sex-ratio imbalances) could limit our identification strategy, because they allow parents to influence the sex of their first child. Similarly, a first-born girl might increase the likelihood of sex-selective abortions in consecutive pregnancies. Using data from the 1991, 2001 and 2011 censuses in India,^42^ we stratified our main regression analysis by state-level sex ratio at birth (children <1 year of age) using the following strata that we observe across Indian states: ≥950 girls per 1000 boys (natural sex ratio at birth),^44^ 900–949 girls per 1000 boys (lower-than-expected sex ratio) and <900 girls per 1000 boys (much-lower-than-expected sex ratio). We attributed sex ratios at birth from the three different censuses to mothers according to the year of their first birth. For each year between the three time points, we calculated sex ratios using linear interpolation. In an additional analysis, we added the state-level sex ratio at birth as a continuous covariate to our main model. Lastly, we conducted an analysis including fixed effects for the 28 508 primary sampling units of our sample to control for any unobserved confounding occurring at the level of the primary sampling unit, such as local child sex preferences or sex ratios.

### Sensitivity analyses

We conducted several sensitivity analyses to further increase confidence in the robustness of our findings. First, we used an alternative definition of our exposure. Specifically, we only included children in a woman’s birth history who were alive at the time of the interview to rule out incomplete exposure to child rearing. Second, we added further socio-demographic covariates to our model. In addition to our main covariates of age, wealth and educational attainment, we included area of residency (urban vs rural), religion (Hindu, Muslim, Christian, Sikh, Buddhist, no religion, other religion) and literacy (cannot read, can read parts of sentences, can read full sentences, not assessable). Third, we added a constant of 10 mmHg to systolic and 5 mmHg to diastolic blood pressure in the 4.0% of mothers in our sample reporting the use of antihypertensive medication, in order to account for the effect of those drugs on blood pressure. This approach is in line with recommendations on adjusting for hypertensive treatment in quantitative data.^45^ Fourth, for each mother, we omitted the first blood pressure measurement and only used the second and third measurements for the calculation of individual blood pressure, as suggested in some of the prior literature.^46^ Fifth, we restricted our sample to mothers who completed their family planning as per self-reported personal preference, sterilization or infecundity (*N* = 352 779). As some mothers with one child might have pursued second pregnancies after data collection, they would have been falsely attributed to the compliers or non-compliers. Sixth, given the large effect of age on blood pressure, we allowed for non-linearities in the relationship of age and blood pressure. To achieve this, we used restricted cubic splines, placing knots at ages 22, 28, 34, 40 and 48 years, which are, respectively, the 0.05, 0.275, 0.5, 0.725 and 0.095 quantiles of the data distribution. This approach allows the relationship between age and blood pressure to take a unique cubic form between each pair of knots with linear relationships below age 22 years and above age 48 years. Seventh, we controlled for previous or current use of oral contraceptives, because the use of oral contraceptives might relate to both the sex of the first-born child and maternal blood pressure. Eighth, we repeated our main analyses without covariates. Ninth, previous longitudinal evidence hints at a potential concave shape in the association of the number of children and maternal blood pressure.^21^ Therefore, we transformed our outcome variables into their natural logarithms and repeated our analyses. Tenth, we added fixed effects for each woman’s respective interviewer to our main model to control for potential measurement error due to systematic differences in blood pressure measurements across interviewers.

All analyses were performed in Stata 15.0.

## Results

### Descriptive statistics

On average, the mothers in our study population were 34.4 years old [standard deviation (SD): 8.1] and had 2.7 children (SD: 1.5) and blood pressures of 116.4 mmHg systolic (SD: 14.4) and 78.5 mmHg diastolic (SD: 9.4). About one in seven mothers (14.7%) was hypertensive, i.e. her systolic blood pressure was ≥140 mmHg, her diastolic blood pressure ≥90 mmHg^47^ or she had received antihypertensive medication. Hypotension, defined as systolic blood pressure <90 mmHg or diastolic blood pressure <60 mmHg, was measured in 1.8% of mothers. Table 1 presents the mean numbers of children and blood pressure levels by various characteristics. Mothers with higher education and wealth, better literacy and urban residency had fewer children, whereas older women had more children. Of the different religions, Muslim mothers reported the highest mean number of children. Blood pressure was higher at older age and lower with lower education and literacy. Individuals identifying as Sikh had the highest mean systolic and diastolic blood pressures compared with followers of the other religions.

**Table 1:** Sample characteristics

Characteristics	Sample size	Number of children, mean	Systolic BP, mean	Diastolic BP, mean
**Age (years)**				
15–24	55 117	1.5	110.3	73.8
25–34	167 583	2.4	113.3	77.0
35–44	153 940	3.1	118.8	80.3
45–49	67 971	3.5	123.4	82.0
**Education**				
None	165 109	3.5	117.7	79.1
Incomplete primary	32 020	2.8	117.5	79.3
Complete primary	35 423	2.7	116.6	78.7
Incomplete secondary	148 433	2.2	115.6	78.2
Complete secondary	29 558	1.9	114.6	77.4
Higher	34 068	1.7	114.1	77.4
**Wealth quintile**				
Poorest	88 119	3.4	116.2	77.8
Poorer	96 335	2.9	116.1	78.1
Middle	92 998	2.6	115.9	78.3
Richer	86 645	2.4	116.5	78.9
Richest	80 514	2.1	117.2	79.3
**Religion**				
Hindu	337 499	2.6	116.1	78.3
Muslim	55 124	3.2	117.4	79.2
Christian	30 650	2.4	116.9	78.6
Sikh	9943	2.3	121.6	81.0
Buddhist	5583	2.5	114.5	78.9
No religion	250	2.7	117.2	80.3
Other	5562	2.6	116.7	79.4
**Residency**				
Urban	124 866	2.4	116.3	79.0
Rural	319 745	2.8	116.4	78.3
**Literacy**				
Not at all	181 975	3.4	117.6	79.1
Parts of sentences	30 901	2.8	117.1	79.0
Full sentences	227 622	2.1	115.4	78.0
Not assessable	3718	3.2	116.6	78.7
**Pooled**	444 611	2.7	116.4	78.5

Estimates calculated using sampling weights as provided in the data set. BP, blood pressure.

**Figure 1:**
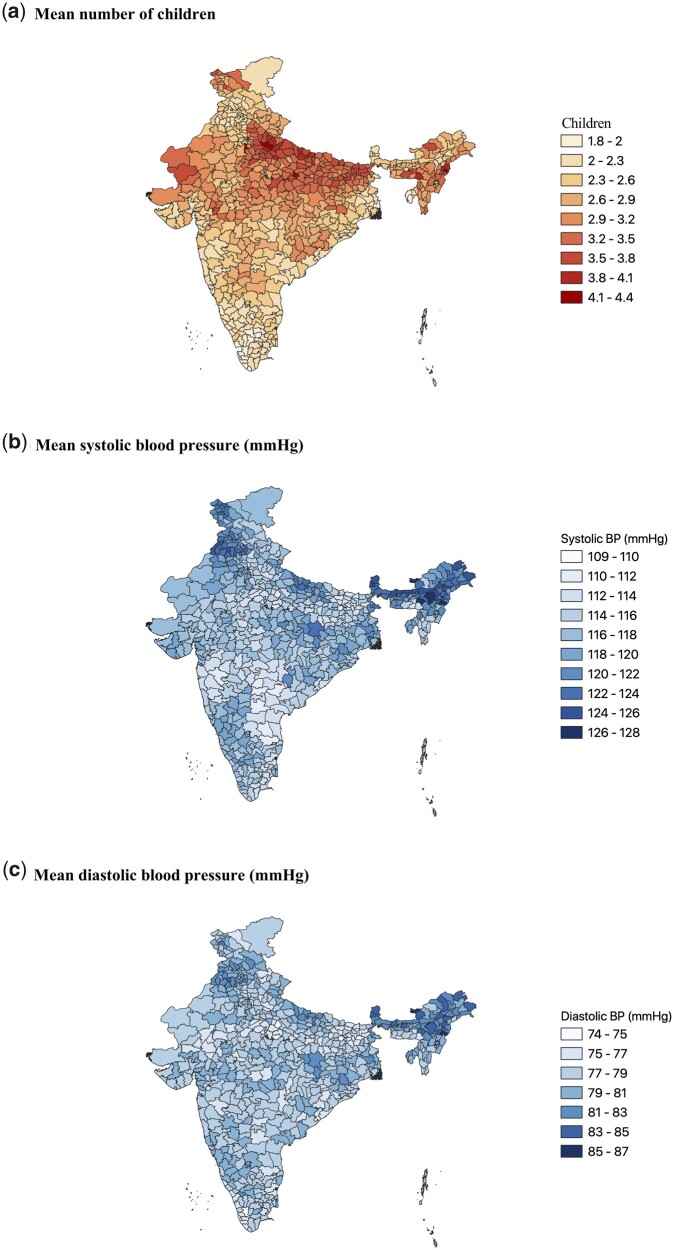
Geographic distribution of exposure and outcomes. Mean number of children (a), systolic (b) and diastolic (c) blood pressure among mothers in our sample across all 640 districts from the 2011 Census in India. Estimates were calculated using sampling weights. For visualization purposes, we chose discrete cut-offs of 0.3 children or 2 mmHg, respectively. BP, blood pressure.

Illustrating the geographic distribution of our exposure and outcomes (Figure 1), we found high mean numbers of children accumulating in areas of the states of Uttar Pradesh and Bihar in the North of India, and low mean numbers of children in South India. Mean blood pressure appeared somewhat higher in parts of Northeast India, and in and around the state of Punjab in the North. Moran’s I indicated that the number of children is spatially clustered at the district level (*P < *0.001), but systolic blood pressure (*P = *0.287) and diastolic blood pressure (*P = *0.509) are not.

### Causal effect of bearing and rearing a child on maternal blood pressure

The first stage of the 2SLS regression shows that mothers with first-born daughters on average had 0.31 more children (95% CI: 0.31 to 0.32, *P *<* *0.001) compared with mothers with first-born sons (Table 2). The corresponding F-statistic was 6544 and hence well above the commonly used cut-off value of 10 for a strong IV.^48^ The central result of our study is reported in the second stage of the 2SLS regression: each additional child beyond the first caused a decrease in both systolic blood pressure [–1.00 mmHg (95% CI: –1.26 to –0.74, *P *<* *0.001)] and diastolic blood pressure [–0.35 mmHg (95% CI: –0.52 to –0.17, *P *<* *0.001)]. The OLS and reduced-form regressions support this finding, revealing effect size estimates in the same direction. These results remained essentially the same across all sensitivity analyses (Supplementary Tables S2–S11, available as Supplementary data at *IJE* online).

**Table 2:** Main regression results

	Systolic blood pressure	Diastolic blood pressure
OLS^a^	–0.42	–0.13
	(–0.46, –0.39)	(–0.15, –0.11)
	[<0.001]	[<0.001]
First stage^b^	0.31	0.31
	(0.31, 0.32)	(0.31, 0.32)
	[<0.001]	[<0.001]
	{6544}	{6544}
Second stage^c^	–1.00	–0.35
	(–1.26, –0.74)	(–0.52, –0.17)
	[<0.001]	[<0.001]
Observations	444 611	

All regression models included age, education categories and wealth quintiles as covariates. Blood pressure was measured in mmHg. The instrumental variable was coded as a binary variable (0 = first child is boy; 1 = first child is girl). 95% confidence intervals in parentheses; *P-*values in square brackets; F-statistics in braces.

a Ordinary least squares regression of maternal blood pressure on number of children.

b First stage of the two-stage least squares regression: number of children regressed on the instrumental variable.

c Second stage of the two-stage least squares regression: maternal blood pressure regressed on the predicted number of children.

### Effect heterogeneity

**Figure 2:**
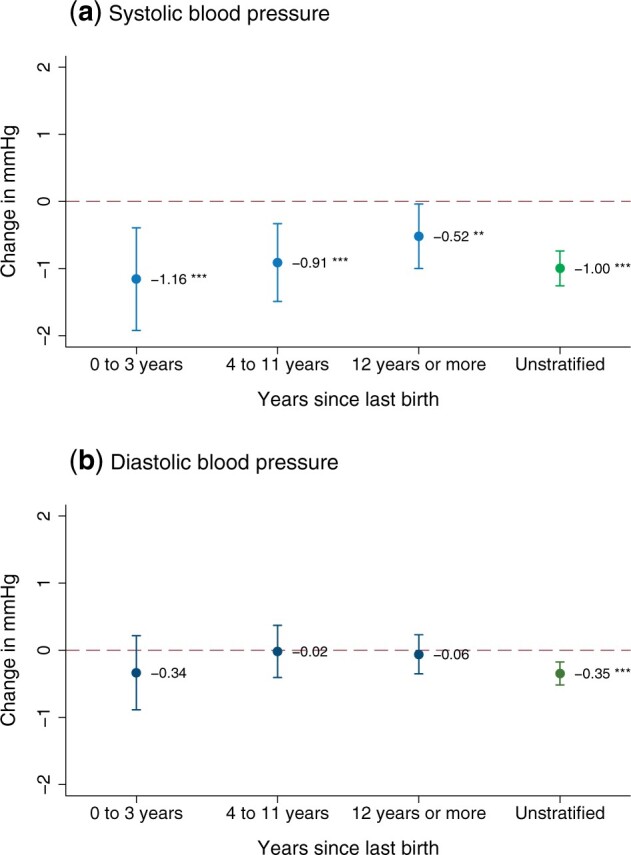
Heterogeneity by years since last birth. The figure shows point estimates and 95% confidence intervals for the causal effect of each child on maternal blood pressure (second stage of the two-stage least squares regression), stratified by time since last birth. The sample was divided into three groups of equal size according to the number of months passed. Group cut-offs were rounded to the closest year. Unstratified main effects are shown in green. Age, years of education and wealth quintiles were included as covariates. ***P <0.01; **P <0.05.

Figure 2 and Supplementary Table S12, available as Supplementary data at *IJE* online, show that systolic blood pressure estimates remained lower than zero in the different time periods since a mother last gave birth but declined slowly over time. Each additional child decreased systolic blood pressure by –1.16 mmHg (95% CI: –1.92 to –0.39, *P *=* *0.002), –0.91 mmHg (95% CI: –1.49 to –0.33, *P *=* *0.003) and –0.52 mmHg (95% CI: –1.00 to –0.04, *P *=* *0.027), respectively, in the periods 0–3, 4–11 and ≥12 years since the last birth. The diastolic blood pressure estimates mostly did not differ from zero.

### Results in sample of men

A total of 53 605 men were included in a separate IV analysis (Table 3). The first stage of the 2SLS regression indicates that husbands/partners of wives with first-born daughters on average had 0.31 more children (95% CI: 0.28 to 0.33, *P *<* *0.001) compared with couples with first-born sons. As the second stage of the 2SLS regression reveals, the average effect of each additional child beyond the first one was 0.05 (95% CI: –0.72 to 0.81, *P *=* *0.900) for systolic and 0.14 (95% CI: –0.40 to 0.67, *P *=* *0.621) for diastolic blood pressure.

**Table 3:** Regression results in men

	Systolic blood pressure	Diastolic blood pressure
OLS ^a^	–0.39	–0.14
	(–0.48, –0.30)	(–0.21, –0.08)
	[<0.001]	[<0.001]
First stage^b^	0.30	0.30
	(0.28, 0.33)	(0.28, 0.33)
	[<0.001]	[<0.001]
	{713}	{713}
Second stage^c^	0.05	0.14
	(–0.72, 0.81)	(–0.40, 0.67)
	[0.900]	[0.621]
Observations	53 605	

All regression models included age, education categories and wealth quintiles as covariates. Blood pressure was measured in mmHg. The instrumental variable was coded as a binary variable (0 = first child is boy; 1 = first child is girl). 95% confidence intervals in parentheses; *P-*values in square brackets; F-statistics in braces.

a Ordinary least squares regression of paternal blood pressure on number of children.

b First stage of the two-stage least squares regression: number of children regressed on the instrumental variable.

c Second stage of the two-stage least squares regression: paternal blood pressure regressed on the predicted number of children.

### Support for the instrumental variable assumptions

**Figure 3:**
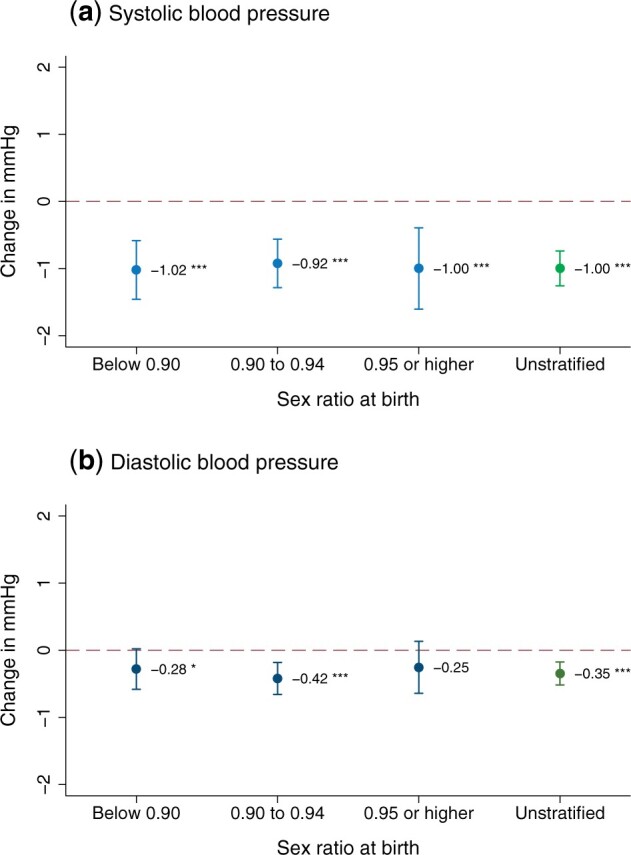
Heterogeneity by state-level sex ratio at birth. The figure shows point estimates and 95% confidence intervals for the causal effect of each child on maternal blood pressure (second stage of the two-stage least squares regression), stratified by state-level sex ratio at birth (children <1 year of age) from the 1991, 2001 and 2011 censuses. Sex ratios for each year between these three time points were calculated using linear interpolation. The sample was divided into three groups using the cut-offs of 900 and 950 girls per 1000 boys. At the time of their first birth, 124 322 mothers resided in states with a sex ratio at birth below 0.90, 176 413 in states with a sex ratio of 0.90–0.95 and 143 876 in states with a sex ratio ≥0.95 (natural ratio). Unstratified main effects are shown in green. Age, years of education and wealth quintiles were included as covariates. ***P <0.01; **P <0.05; *P <0.1.

Supplementary Table S13, available as Supplementary data at *IJE* online, shows that the observed characteristics were nearly perfectly balanced across the two values of our IV, supporting the exclusion-restriction assumption of our analysis. Next, we stratified our analysis by state-level sex ratio at birth. In our sample, at the time of their first birth, 124 322 mothers resided in states with a sex ratio at birth of <900 girls per 1000 boys, 176 413 in states with a ratio of 900–949 girls per 1000 boys, and 143 876 in states with a ratio of ≥950 girls per 1000 boys. The 2SLS regression results in all three strata were similar to our unstratified main results (Figure 3 and Supplementary Table S14, available as Supplementary data at *IJE* online). The main results were also robust to adding state-level sex ratio at birth as a continuous covariate to the model (Supplementary Table S15, available as Supplementary data at *IJE* online) and to including fixed effects for the 28 508 primary sampling units of our sample (Supplementary Table S16, available as Supplementary data at *IJE* online). Considering these findings, we concluded that sex-selective abortions are unlikely to substantially affect our results. Taken together, these tests further confirm that the IV assumptions are likely met in our study (Supplementary Text S1, available as Supplementary data at *IJE* online).

## Discussion

Our results suggest that bearing and rearing a child causes a decrease in blood pressure in Indian mothers. Each child beyond the first lowered systolic blood pressure by ∼1 mmHg and diastolic blood pressure by ∼1/3 mmHg. The effect on systolic blood pressure waned over time, yet remained lower than zero even more than a decade after childbirth. Although the effect sizes might be considered small in a clinical context, at the population level, they are substantial,^49^ particularly as blood pressure is log-linearly linked to CVD risk without any threshold.^50^ We did not observe comparable effects among men, indicating that behaviour changes that an additional child induces in both women and men are not responsible for the observed causal effects on blood pressure. Rather, it is likely that the biological and behavioural changes that child bearing and rearing specifically induce in women explain the effects.

Our findings are important for population health and policy. First, they mitigate common concerns that bearing and rearing a child could increase hypertension and CVD risk among mothers in the long term.^11^ Such concerns are mainly based on the striking short-term effect of pregnancy on maternal blood pressure.^2^ Our results suggest that this increase does not persist and is even reversed beyond delivery.

Second, at the population level, women have lower blood pressure than men, in India as well as globally.^7^^,^^51^ According to our results, one source of this female blood pressure advantage is child bearing and rearing. As fertility continues to decline in India and similar countries,^5^^,^^52^ this advantage may shrink. Policy makers should thus pay increased attention to hypertension prevention targeted at women. Traditionally, CVD prevention and care have tended to target men.^53–55^ Such prevention programmes will likely have to be redesigned and specifically tailored to meet women’s needs and expectations.

Third, pregnant women worldwide nearly universally attend antenatal care at least once, where they are nearly universally screened for hypertension.^56^^,^^57^ In fact, antenatal care is one of the few prevention programmes in low- and middle-income countries that achieve such high levels of coverage of their target population.^57–59^ Blood pressure screening in antenatal care will detect women with high blood pressure both due to and independently of their pregnancies. According to our results, however, women who do not have children could be particularly predisposed to hypertension. In India, 5–10% of women do not experience any pregnancy throughout their lifetime and hence miss blood pressure screening during antenatal care.^60^^,^^61^ In our sample, 90% of mothers had their blood pressure measured during their last pregnancy. Novel health programmes are likely needed to reach the population of women without children with blood pressure screening offerings. Opportunities to enhance blood pressure screening and prevention for women—and in particular for women without children—include large-scale community-based screening programmes;^7^ integration of blood pressure screening into other healthcare services targeted at women, such as cervical-cancer screening^62^ and family planning;^63^ and screening by community health workers, such as the accredited social health activists (ASHAs)^64^ in India, who focus on women’s health during their regular home visits.^65^

Whereas some longitudinal studies suggest an inverse association between having children and maternal blood pressure,^19–21^ others show higher blood pressure among women with more children.^22^ Similarly, an inverse association is seen in some,^12–15^ but not all,^16–18^ cross-sectional studies. However, previous studies were likely prone to confounding by several biologic,^27^^,^^28^ socioeconomic^29^^,^^30^ and behavioural factors,^66^^,^^67^ as well as selection effects. By using an IV approach in a large-scale nationally representative data set comprising >400 000 Indian mothers, we have likely overcome these limitations of the previous literature. For instance, stress negatively impacts fertility^66^ and increases hypertension risk.^67^ Hence, it is possible that stress confounds the relationship between children and maternal blood pressure. However, stress has not been measured and controlled for in past studies.^12–21^ Our IV approach controls for stress and other sources of unobserved confounding.^23^^,^^31^^,^^32^

Replication of our quasi-experimental study in other populations with son preference would be desirable to evaluate whether the observed effects are specific to India or also apply to other countries. Pregnancy and motherhood are major events in a woman’s life. Hence, they can affect blood pressure through various pathways that might explain our findings in this study and also apply in other contexts. Whereas blood pressure increases towards delivery,^2^ during the postpartum period hemodynamic adaptations are to a large extent reversed to the pre-pregnancy state.^1^ Some cardiovascular parameters even appear to be favourably influenced by the hyperdynamic stimuli imposed by pregnancy: reductions in vascular resistance and arterial stiffness, for instance, can be observed over at least a year after delivery.^68^^,^^69^ Additionally, along the months or years after delivery, most Indian mothers breastfeed their children.^70^ Breastfeeding is associated with lower maternal blood pressure and lower hypertension risk,^71^ a relationship that is potentially mediated through hormonal and metabolic changes occurring during lactation.^72^ Current or recent breastfeeding might explain why we found a larger blood pressure-reducing effect of children in mothers with more recent births.^73^

Behaviourally, children can act as a strong motivation for mothers to improve certain health behaviours, such as ceasing smoking.^74^ Up to 70% of women are estimated to quit smoking while pregnant.^75^ Whereas about a quarter of women who used to smoke persistently remain abstinent after pregnancy, relapse rates among the others increase over time after delivery.^76^ Such a behavioural dynamic could explain the effect of having children on maternal blood pressure waning over time after childbirth. In general, women experience vast lifestyle changes while raising children. For instance, child rearing might increase physical activity in daily life, contributing to blood pressure reductions. Other plausible mechanisms causally connecting child rearing to blood pressure may be more structural and differ across contexts. For instance, children require resources and may thus deteriorate the economic situation and availability of calories in poorer households, leading to blood pressure reduction via weight loss.

The absence of an effect of additional children on blood pressure in men underscores that our findings for women are likely not due to behavior changes affecting all household members and that underlying pathways are sex-specific. In the Indian context, men tend to take on different roles in raising children compared with women.^77^ Therefore, plausible women-specific pathways comprise not only biological factors such as breastfeeding, but also maternal behaviour changes not commonly observed among Indian fathers.

Our study has several strengths. We estimated the effect of children on maternal blood pressure for the first time in a nationally representative data set. We do so for India, which is projected to overtake China as the world’s most populous country by 2027.^52^ Importantly, our quasi-experimental approach allows us to answer our research question controlling for both observed and unobserved confounding. Because of the strong effect of our IV on the exposure, which is highly plausible given the pervasive son preference in India,^34^^,^^35^ and the strong theoretical support for the assumption that the IV does not independently affect blood pressure, our analysis is likely to have generated valid effect size estimates.^31^^,^^41^^,^^48^ Several statistical tests further support the validty of the IV approach in this study.

Our study also has several limitations. First, the birth history variables in our data set might underreport pregnancies, because the NFHS-4 questionnaire did not explicitly ask for stillbirths and miscarriages. For the same reason, it was also not possible to differentiate between stillbirths and neonatal deaths occurring on the day of delivery.^78^ Second, in the absence of longitudinal data, we could only approximate time trends by comparing blood pressure at different time intervals since mothers last gave birth. The results presented in Figure 2 might therefore not depict longitudinal trends with full precision. Third, as described above, our IV analysis cannot provide information on first-born children, which might have an even stronger effect on maternal blood pressure.^20^^,^^21^ Similarly, we cannot draw conclusions about women who were nulliparous at the time of data collection, e.g. due to infertility, recurrent miscarriages or young age. Nevertheless, a key strength of our instrument is that it affected a large segment of the population, as reported in the first-stage regression. Fourth, our sample is nationally representative within the study’s age range and thus the 2SLS regression results are average effects across the nationally representative points in women's birth histories rather than average effects across women's full life-time birth histories. However, our results proved robust when we conducted our analysis in the subsample of women who had finished their family planning. Fifth, despite the relevance of population-level decreases in blood pressure for CVD risk,^49^^,^^50^ and the early onset of CVD among Indians,^8^^,^^9^ we cannot empirically prove that the observed effects persist beyond the upper age limit of our study population (49 years of age). Sixth, the sample of men in the NFHS-4 was much smaller than the sample of women, implying substantially lower statistical power to detect signficant effects. Given that the point estimates of the insignificant effects in men are close to zero, however, it is not very likely that large significant effects would have emerged with larger samples. Lastly, whereas violations of the IV assumptions appear unlikely in our study, it is impossible to prove that they are definitively met.^38^

In conclusion, bearing and rearing a child decreases blood pressure among mothers in India. As fertility continues to decline in India and similar countries, policy makers should design and implement hypertension screening and prevention programmes that specifically target women.

## Supplementary data

Supplementary data are available at *IJE* online.

## Ethics approval

This study used an anonymized publicly available data set with no identifiable information on the survey participants, and thus did not require ethics approval.

## Funding

T.B. was supported by the Alexander von Humboldt Foundation through the Alexander von Humboldt Professor award, funded by the German Federal Ministry of Education and Research. T.B. also received funding from the Wellcome Trust under Award Number 208766/Z/17/Z. J.W.D.N. was supported by the Alexander von Humboldt Foundation. P.G. was supported by the National Center for Advancing Translational Sciences of the National Institutes of Health under Award Number KL2TR003143. F.T. was supported by the Else Kröner-Fresenius-Foundation within the Heidelberg Graduate School of Global Health at the Heidelberg Institute of Global Health, Heidelberg University. The funders had no role in study design, data collection and analysis, decision to publish or preparation of the manuscript.

## Data availability

The NFHS-4 survey data are publicly available and distributed through the website of the Demographic and Health Surveys (DHS).

## Supplementary Material

dyab058_Supplementary_Data
