# Supplementary material for: The effect of bearing and rearing a child on blood pressure: a nationally representative instrumental variable analysis of 444 611 mothers in India
Source: Int J Epidemiol. 2021 Jul 19;50(5):1671–83. doi: 10.1093/ije/dyab058 (PMC8580275; doi:10.1093/ije/dyab058)
Supplement: dyab058_Supplementary_Data [file dyab058_supplementary_data.docx]

**SUPPLEMENTARY MATERIAL**

[Supplementary Methods: Model equations 2](#_Toc65177758)

[Text S1: Instrumental variable assumptions 3](#_Toc65177759)

[Figure S1. Flow diagram of study population. 5](#_Toc65177760)

[Figure S2. Directed acyclic graph (DAG) of instrumental variable (IV) study design. 6](#_Toc65177761)

[Table S1. Regression results including reduced form estimates. 7](#_Toc65177762)

[Table S2. Sensitivity analysis: living children only. 8](#_Toc65177763)

[Table S3. Sensitivity analysis: including further sociodemographic covariates 9](#_Toc65177764)

[Table S4. Sensitivity analysis: correcting for blood pressure medication. 10](#_Toc65177765)

[Table S5. Sensitivity analysis: different calculation of blood pressure. 11](#_Toc65177766)

[Table S6. Sensitivity analysis: finished family planning. 12](#_Toc65177767)

[Table S7. Sensitivity analysis: using restricted cubic splines for age. 13](#_Toc65177768)

[Table S8. Sensitivity analysis: oral contraceptive use. 14](#_Toc65177769)

[Table S9. Sensitivity analysis: model without covariates. 15](#_Toc65177770)

[Table S10. Sensitivity analysis: ln-transformed outcome. 16](#_Toc65177771)

[Table S11. Sensitivity analysis: interviewer fixed effects 17](#_Toc65177772)

[Table S12. Heterogeneity by years since last birth. 18](#_Toc65177773)

[Table S13. Observed characteristics across instrument levels. 19](#_Toc65177774)

[Table S14. Heterogeneity by state-level sex ratio at birth. 20](#_Toc65177775)

[Table S15. Sensitivity analysis: state-level sex ratio at birth 21](#_Toc65177776)

[Table S16. Sensitivity analysis: Primary sampling unit fixed effects 22](#_Toc65177777)

[References for Appendix 23](#_Toc65177778)

# Supplementary Methods: Model equations

*OLS regression :* ${BP}_{i}=\alpha_{0}+\alpha_{1}{n\_children}_{i}+ \alpha_{ij}X_{ij}+ \varepsilon_{i}$ *(1)*

${BP}_{i}$: Continuous maternal systolic or diastolic blood pressure of individual i.

$\alpha_{0}$: Intercept

$\alpha_{1}$: Coefficient for number of children ${n\_children}_{i}$

$\alpha_{ij}$: Coefficient for covariate $X_{j}$ of individual i.

$\varepsilon_{i}$: Residual for individual i.

*First stage regression:* ${n\_children}_{i}=\beta_{0}+\beta_{1}IV+ \beta_{ij}X_{ij}+ \varepsilon_{i}$ *(2)*

$\beta_{1}$: Coefficient of the instrumental variable $IV$ (=1 if female first-born; =0 if male)

*Second stage regression:* ${BP}_{i}=\gamma_{0}+\gamma_{1}\hat{{n\_children}_{i}}+ \gamma_{ij}X_{ij}+ \varepsilon_{i}$ *(3)*

$\hat{{n\_children}_{i}}$ Predicted number of children of mother i from first stage regression.

$\gamma_{1}$: Complier average causal effect

*Reduced form regression:* ${BP}_{i}=\delta_{0}+\delta_{1}IV+ \delta_{ij}X_{ij}+ \varepsilon_{i}$ *(4)*

$\delta_{1}$: Intention-to-treat effect

# Text S1: Instrumental variable assumptions

The crucial advantage of the instrumental variable approach is that, as long as the assumptions for the instrument being valid are met, it is vulnerable to neither observed nor unobserved confounding, and thus allows causal inference (Maciejewski & Brookhart, 2019; Hernan & Robins, 2006, Martens et al., 2006).

There are three key instrumental variable assumptions: the relevance assumption, the exclusion restriction assumption, and the exchangeability assumption (Lousdal, 2018). The *relevance assumption*, which states that the instrument must have a sufficient effect on the exposure, is empirically verified by the large F statistic (6544) from the first stage regression. The plausibility of the other two conditions being met can be ascertained by thorough reasoning and supporting empirical tests (Bärnighausen, 2017).

The *exclusion restriction assumption* states that the instrument must only affect the outcome through the exposure of interest. Applied to our study, the fact that the first-born child is a girl must not influence maternal blood pressure other than through the total amount of children her mother will have. As a conceivable limitation, a female fetus could yield different hemodynamic implications compared to a male one. While some evidence analyzing pregnancy-induced hypertension might hint in this direction, such an effect, however, has not been shown for blood pressure in general or a broader population, and does not persist beyond delivery (Petry et al., 2014). Similarly, a first-born boy might increase the likelihood of sex-selective abortions in consecutive pregnancies. An association between abortions and increased CVD risk has previously been shown in comparable populations (Peters et al., 2017). We assessed this potential limitation by the analyses described in the next paragraph. Lastly, a first-born son may result in a higher chance for the mother taking oral contraceptives to prevent subsequent pregnancies, which might lead to increased blood pressure. Controlling for the use of oral contraceptives, however, essentially did not change the results of the 2SLS regression.

The *exchangeability assumption* requires the instrument to not share common causes with the outcome. Here, the sex of the first child may not root in factors that relate to maternal blood pressure. In this context, sex-selective abortions need to be discussed. Pre-natal diagnostics and abortions allow parents to influence the sex of their offspring and are associated with various maternal characteristics potentially related to blood pressure, most prominently higher wealth and education (Jha et al., 2011; Bhalotra & Cochrane, 2010; Pörtner, 2010). Similarly, first-born girls who died due to neglect or infanticide might remain unreported. As we observed sex ratio imbalance among first-born children in our dataset (Table S13), we quantitatively explored this potential limitation. When stratifying our analysis by state-level sex ratio at birth, we observed similar effect sizes across strata and in comparison to our main results (Figure 3 and Table S14): -1.02 mmHg (95% confidence interval: -1.46 to -0.58, *P*<0.001, n = 124 322) systolic blood pressure for mothers residing in states with sex ratios below 0.90 girls per boy, -0.92 mmHg (95% confidence interval: -1.28 to -0.56, *P*<0.001, n = 176 413) in states with ratios between 0.90 and 0.95, and -1.00 mmHg (95% confidence interval: -1.60 to -0.39, *P*=0.001, n = 143 876) in states with ratios of 0.95 or above (natural ratio). Additionally, we found that observed maternal characteristics, including wealth and education, were virtually the same for women with a first-born girl as they were for women with a first-born boy (Table S13). Thus, it appears unlikely that sex-selective abortions in first pregnancies limit the exchangeability assumption. Similarly, sex-selective abortions in consecutive pregnancies are unlikely to limit the exclusion restriction assumption. In conclusion, the instrumental variable assumptions appear to be satisfied in our study.

Apart from these three conditions, there is a fourth assumption, which is needed in order to identify point estimates as opposed to only effect bounds (Lousdal, 2018). This so-called monotonicity assumption sets the prerequisite that there be no “defiers” in the sample. In our study, defiers are women who would not pursue an additional childbirth after a first-born girl, but would do so after giving birth to a boy. While such a strong girl preference is conceivable at the individual level, the Indian cultural context makes an aversion towards male offspring on a larger scale highly unlikely (Almond et al., 2013; Pande & Astone, 2007).

# Figure S1. Flow diagram of study population.

Flow diagram illustrating the exclusion criteria applied to construct our study population. Share of individuals lost at each stage in relation to previous stage noted as percentages in brackets. The India National Family and Health Survey (NFHS-4) yielded a household response rate of 96%.

Figure S2. Directed acyclic graph (DAG) of instrumental variable (IV) study design. DAG illustrating the methodologic concept of our IV analysis. Dotted lines represent excluded associations.

Table S1. Regression results including reduced form estimates. Point estimates of the regression analyses. All regression models included age, educational attainment, and wealth quintiles as covariates. Blood pressure was measured in mmHg. The instrument was coded as a binary variable (0 = first child is boy; 1 = first child is girl). 95% confidence interval in parentheses; P values in square brackets; F-statistic in braces.

† Ordinary least squares regression of maternal blood pressure on number of children.

‡ Reduced form regression of maternal blood pressure on the instrumental variable.

§ First stage of the two-stage least squares regression: the number of children on the instrumental variable.

‖ Second stage of the two-stage least squares regression: maternal blood pressure on the predicted number of children.

Table S2. Sensitivity analysis: living children only. Point estimates of the regression analyses. All regression models included age, educational attainment, and wealth quintiles as covariates. Only children alive at the time of the interview were included in women’s birth histories. Blood pressure was measured in mmHg. The instrument was coded as a binary variable (0 = first child is boy; 1 = first child is girl). 95% confidence interval in parentheses; P values in square brackets; F-statistic in braces.

† Ordinary least squares regression of maternal blood pressure on number of children.

‡ First stage of the two-stage least squares regression: the number of children on the instrumental variable.

§ Second stage of the two-stage least squares regression: maternal blood pressure on the predicted number of children.

Table S3. Sensitivity analysis: including further sociodemographic covariates**.** Point estimates of the regression analyses. All regression models included age, educational attainment, wealth quintiles, religion, area of residency and literacy as covariates. Blood pressure was measured in mmHg. The instrument was coded as a binary variable (0 = first child is boy; 1 = first child is girl). 95% confidence interval in parentheses; P values in square brackets; F-statistic in braces.

† Ordinary least squares regression of maternal blood pressure on number of children.

‡ First stage of the two-stage least squares regression: the number of children on the instrumental variable.

§ Second stage of the two-stage least squares regression: maternal blood pressure on the predicted number of children.

Table S4. Sensitivity analysis: correcting for blood pressure medication. Point estimates of the regression analyses adding 10 / 5 mmHg to blood pressure measurements in mothers that receive blood pressure medication. All regression models included age, educational attainment and wealth quintiles as covariates. Blood pressure was measured in mmHg. The instrument was coded as a binary variable (0 = first child is boy; 1 = first child is girl). 95% confidence interval in parentheses; P values in square brackets; F-statistic in braces.

† Ordinary least squares regression of maternal blood pressure on number of children.

‡ First stage of the two-stage least squares regression: the number of children on the instrumental variable.

§ Second stage of the two-stage least squares regression: maternal blood pressure on the predicted number of children.

Table S5. Sensitivity analysis: different calculation of blood pressure. Point estimates of the regression analyses using the average of the second and third blood pressure measurement of each woman. Blood pressure was measured in mmHg. All regression models included age, educational attainment and wealth quintiles as covariates. The instrument was coded as a binary variable (0 = first child is boy; 1 = first child is girl). 95% confidence interval in parentheses; P values in square brackets; F-statistic in braces.

† Ordinary least squares regression of maternal blood pressure on number of children.

‡ First stage of the two-stage least squares regression: the number of children on the instrumental variable.

§ Second stage of the two-stage least squares regression: maternal blood pressure on the predicted number of children.

Table S6. Sensitivity analysis: finished family planning. Point estimates of the regression analyses excluding mothers that reported to have not finished their family planning yet. All regression models included age, educational attainment and wealth quintiles as covariates. Blood pressure was measured in mmHg. The instrument was coded as a binary variable (0 = first child is boy; 1 = first child is girl). 95% confidence interval in parentheses; P values in square brackets; F-statistic in braces.

† Ordinary least squares regression of maternal blood pressure on number of children.

‡ First stage of the two-stage least squares regression: the number of children on the instrumental variable.

§ Second stage of the two-stage least squares regression: maternal blood pressure on the predicted number of children.

Table S7. Sensitivity analysis: using restricted cubic splines for age. Point estimates of the regression analyses allowing for non-linearities in the effect of age on blood pressure using restricted cubic splines. Additionally, all regression models included educational attainment and wealth quintiles as covariates. Blood pressure was measured in mmHg. The instrument was coded as a binary variable (0 = first child is boy; 1 = first child is girl). 95% confidence interval in parentheses; P values in square brackets; F-statistic in braces.

† Ordinary least squares regression of maternal blood pressure on number of children.

‡ First stage of the two-stage least squares regression: the number of children on the instrumental variable.

§ Second stage of the two-stage least squares regression: maternal blood pressure on the predicted number of children.

Table S8. Sensitivity analysis: oral contraceptive use. Point estimates of the regression analyses. All regression models included age, education categories, wealth quintiles, and the current or previous use of oral contraceptives as covariates (0=never used oral contraceptives, 1=used oral contraceptives before). Blood pressure was measured in mmHg. The instrument was coded as a binary variable (0 = first child is boy; 1 = first child is girl). 95% confidence interval in parentheses; P values in square brackets; F-statistic in braces.

† Ordinary least squares regression of maternal blood pressure on number of children.

‡ First stage of the two-stage least squares regression: the number of children on the instrumental variable.

§ Second stage of the two-stage least squares regression: maternal blood pressure on the predicted number of children.

Table S9. Sensitivity analysis: model without covariates. Point estimates of the regression analyses. All regression models did not include any covariates. Blood pressure was measured in mmHg. The instrument was coded as a binary variable (0 = first child is boy; 1 = first child is girl). 95% confidence interval in parentheses; P values in square brackets; F-statistic in braces.

† Ordinary least squares regression of maternal blood pressure on number of children.

‡ First stage of the two-stage least squares regression: the number of children on the instrumental variable.

§ Second stage of the two-stage least squares regression: maternal blood pressure on the predicted number of children.

Table S10. Sensitivity analysis: ln-transformed outcome. Point estimates of the regression analyses. All regression models included age, education categories and wealth quintiles as covariates. Blood pressure was measured in mmHg and transformed to its natural logarithm. Estimates reflect the relative change in the outcome variable across values of the exposure. The instrument was coded as a binary variable (0 = first child is boy; 1 = first child is girl). 95% confidence interval in parentheses; P values in square brackets; F-statistic in braces.

† Ordinary least squares regression of maternal blood pressure on number of children.

‡ First stage of the two-stage least squares regression: the number of children on the instrumental variable.

§ Second stage of the two-stage least squares regression: maternal blood pressure on the predicted number of children.

Table S11. Sensitivity analysis: interviewer fixed effects**.** Point estimates of the regression analyses. All regression models included age, educational attainment, wealth quintiles, and interviewer identification number (categorical) as covariates. Blood pressure was measured in mmHg. The instrument was coded as a binary variable (0 = first child is boy; 1 = first child is girl). 95% confidence interval in parentheses; P values in square brackets; F-statistic in braces.

† Ordinary least squares regression of maternal blood pressure on number of children.

‡ First stage of the two-stage least squares regression: the number of children on the instrumental variable.

§ Second stage of the two-stage least squares regression: maternal blood pressure on the predicted number of children.

Table S12. Heterogeneity by years since last birth. Point estimates of the regression analyses stratified by time since the last birth of a woman. The sample was divided into three equally sized groups according to the number of months passed since last birth, and group cut-offs rounded to the closest year. All regression models included age, years of education and wealth quintiles as covariates. 95% confidence interval in parentheses; *P* values in square brackets.

† Ordinary least squares regression of maternal blood pressure on number of children.

‡ First stage of the two-stage least squares regression: the number of children on the instrumental variable.

§ Second stage of the two-stage least squares regression: maternal blood pressure on the predicted number of children.

Table S13. Observed characteristics across instrument levels. Means and standard deviations (SD) for several variables across levels of the instrument using sampling weights provided in the dataset. The unit of age is years. Categorical variables were translated into discrete numerical values.

Table S14. Heterogeneity by state-level sex ratio at birth. Point estimates of the regression analyses stratified by state-level sex ratio at birth. The sample was divided into three groups using the cut-offs of 900 and 950 girls per 1000 boys. Data on sex ratios at birth (children younger than 1 year of age) from the 1991, 2001 and 2011 census in India. All regression analyses included age, educational attainment and wealth quintiles as covariates. 95% confidence interval in parentheses; *P* values in square brackets.

† Ordinary least squares regression of maternal blood pressure on number of children.

‡ First stage of the two-stage least squares regression: the number of children on the instrumental variable.

§ Second stage of the two-stage least squares regression: maternal blood pressure on the predicted number of children.

Table S15. Sensitivity analysis: state-level sex ratio at birth. Point estimates of the regression analyses. All regression models included age, educational attainment, wealth quintiles, and state-level sex ratio at birth (continuous) as covariates. Data on sex ratios at birth (children younger than 1 year of age) from the 1991, 2001 and 2011 census in India. Blood pressure was measured in mmHg. The instrument was coded as a binary variable (0 = first child is boy; 1 = first child is girl). 95% confidence interval in parentheses; P values in square brackets; F-statistic in braces.

† Ordinary least squares regression of maternal blood pressure on number of children.

‡ First stage of the two-stage least squares regression: the number of children on the instrumental variable.

§ Second stage of the two-stage least squares regression: maternal blood pressure on the predicted number of children.

Table S16. Sensitivity analysis: Primary sampling unit fixed effects**.** Point estimates of the regression analyses. All regression models included age, educational attainment, wealth quintiles, and primary sampling unit as covariates. Blood pressure was measured in mmHg. The instrument was coded as a binary variable (0 = first child is boy; 1 = first child is girl). 95% confidence interval in parentheses; P values in square brackets; F-statistic in braces.

† Ordinary least squares regression of maternal blood pressure on number of children.

‡ First stage of the two-stage least squares regression: the number of children on the instrumental variable.

§ Second stage of the two-stage least squares regression: maternal blood pressure on the predicted number of children.

**References for Supplementary Material**

Almond D, Edlund L, Milligan K. Son preference and the persistence of culture: Evidence from South and East Asian immigrants to Canada. *Population and Development Review*. 2013;39(1):75-95.

Bärnighausen T, Oldenburg C, Tugwell P, et al. Quasi-experimental study designs series-paper 7: Assessing the assumptions. *Journal of Clinical Epidemiology*. 2017;89:53-66.

Bhalotra S, Cochrane T. *Where have all the young girls gone? Identification of sex selection in India:* Institute of Labor Economics (IZA); 2010.

Hernan MA, Robins JM. Instruments for causal inference: An epidemiologist's dream? *Epidemiology*. 2006;17(4):360-72.

Jha P, Kesler MA, Kumar R, et al. Trends in selective abortions of girls in India: Analysis of nationally representative birth histories from 1990 to 2005 and census data from 1991 to 2011. *Lancet*. 2011;377(9781):1921-8.

Lousdal ML. An introduction to instrumental variable assumptions, validation and estimation. *Emerging Themes in Epidemiology*. 2018;15.

Maciejewski ML, Brookhart MA. Using instrumental variables to address bias from unobserved confounders. *JAMA*. 2019;321(21):2124-5.

Martens EP, Pestman WR, de Boer A, Belitser SV, Klungel OH. Instrumental variables: Application and limitations. *Epidemiology*. 2006;17(3):260-7.

Pande RP, Astone NM. Explaining son preference in rural India: The independent role of structural versus individual factors. *Population Research and Policy Review*. 2007;26(1):1-29.

Peters SAE, Yang L, Guo Y, et al. Pregnancy, pregnancy loss, and the risk of cardiovascular disease in Chinese women: findings from the China Kadoorie Biobank. BMC Medicine 2017; 15: 148.

Petry CJ, Beardsall K, Dunger DB. The potential impact of the fetal genotype on maternal blood pressure during pregnancy. *Journal of Hypertension*. 2014;32(8):1553-61.

Pörtner C. *Sex Selective Abortions, Fertility and Birth Spacing:* University of Washington, Department of Economics; 2010.
